# Antibiotics Act with vB_AbaP_AGC01 Phage against *Acinetobacter baumannii* in Human Heat-Inactivated Plasma Blood and *Galleria mellonella* Models

**DOI:** 10.3390/ijms21124390

**Published:** 2020-06-19

**Authors:** Bartłomiej Grygorcewicz, Marta Roszak, Piotr Golec, Daria Śleboda-Taront, Natalia Łubowska, Martyna Górska, Joanna Jursa-Kulesza, Rafał Rakoczy, Bartosz Wojciuk, Barbara Dołęgowska

**Affiliations:** 1 Department of Laboratory Medicine, Pomeranian Medical University in Szczecin, Powstańców Wielkopolskich 72, 70-111 Szczecin, Poland; martaroszak95@gmail.com (M.R.); daria.sleboda@pum.edu.pl (D.Ś.-T.); barbara.dolegowska@pum.edu.pl (B.D.); 2 Department of Molecular Virology, Institute of Microbiology, Faculty of Biology, University of Warsaw, Miecznikowa 1, 02-096 Warsaw, Poland; pgolec@biol.uw.edu.pl; 3 Department of Medical Microbiology, Faculty of Medicine, Medical University of Gdansk; Marii Skłodowskiej-Curie 3a, 80-210 Gdańsk, Poland; nlubowska@gumed.edu.pl; 4 Department of General Pathology, Pomeranian Medical University in Szczecin, Powstańców Wielkopolskich 72, 70-111 Szczecin, Poland; martyna.gorska@pum.edu.pl; 5 Department of Medical Microbiology, Pomeranian Medical University in Szczecin, Powstańców Wielkopolskich 72, 70-111 Szczecin, Poland; asiaju@pum.edu.pl; 6 Institute of Chemical Engineering and Environmental Protection Process, Faculty of Chemical Engineering, West Pomeranian University of Technology, al. Piastów 42, 71-065 Szczecin, Poland; rrakoczy@zut.edu.pl; 7 Department of Diagnostic Immunology, Pomeranian Medical University in Szczecin, Powstańców Wielkopolskich 72, 70-111 Szczecin, Poland; bartosz.wojciuk@pum.edu.pl

**Keywords:** *Acinetobacter baumannii*, antibiotics resistance, bacteriophages, blood infection, *Galleria mellonella*

## Abstract

Increasing multidrug resistance has led to renewed interest in phage-based therapy. A combination of the bacteriophages and antibiotics presents a promising approach enhancing the phage therapy effectiveness. First, phage candidates for therapy should be deeply characterized. Here we characterize the bacteriophage vB_AbaP_AGC01 that poses antibacterial activity against clinical Acinetobacter baumannii strains. Moreover, besides genomic and phenotypic analysis our study aims to analyze phage–antibiotic combination effectiveness with the use of ex vivo and in vivo models. The phage AGC01 efficiently adsorbs to A. baumannii cells and possesses a bacteriolytic lifecycle resulting in high production of progeny phages (317 ± 20 PFU × cell^−1^). The broad host range (50.27%, 93 out of 185 strains) against A. baumannii isolates and the inability of AGC01 to infect other bacterial species show its high specificity. Genomic analysis revealed a high similarity of the AGC01 genome sequence with that of the *Friunavirus* genus from a subfamily of Autographivirinae. The AGC01 is able to significantly reduce the A. baumannii cell count in a human heat-inactivated plasma blood model (HIP-B), both alone and in combination with antibiotics (gentamicin (GEN), ciprofloxacin (CIP), and meropenem (MER)). The synergistic action was observed when a combination of phage treatment with CIP or MER was used. The antimicrobial activity of AGC01 and phage-antibiotic combinations was confirmed using an in vivo larva model. This study shows the greatest increase in survival of G. mellonella larvae when the combination of phage (MOI = 1) and MER was used, which increased larval survival from 35% to 77%. Hence, AGC01 represents a novel candidate for phage therapy. Additionally, our study suggests that phages and antibiotics can act synergistically for greater antimicrobial effect when used as combination therapy.

## 1. Introduction

The rising incidence of multi- and pan-drug resistant bacterial strains poses a severe challenge to medical care worldwide [1,2]. The majority of drug-resistant infection is associated with members of the ESKAPE (*Enterococcus faecium*, *Staphylococcus aureus*, *Klebsiella pneumoniae*, *Acinetobacter baumannii*, *Pseudomonas aeruginosa*, and *Enterobacter* species) group [3]. According to the WHO list of antibiotic-resistant bacteria, carbapenem-resistant *A. baumannii* poses a critical threat to public health, with limited therapeutic options. Ipso facto, the WHO has highlighted the need to prioritize research on the development of new antibiotics and alternative treatment options for the listed drug-resistant bacteria [4]. 

*A. baumannii* causes one of the most challenging nosocomial infections, with mortality rates as high as 35% [5]. This bacterium is responsible for a large spectrum of hospital-acquired infections such as bacteremia, sepsis, urinary tract infection, pneumonia, and burn wound infections, and has developed resistance to almost all known antibiotics [6]. One of the most promising tools in combatting antibiotic-resistant bacteria is the use of bacteriophages, viruses that infect bacteria [7]. 

As a result of widespread antimicrobial drug resistance, alternative treatments such as bacteriophage therapy are gaining increasing interest. Bacteriophages specifically infect and propagate inside of bacterial cells and are promising tools to combat bacterial infection [8]. Phages appear to be safe and neutral to the human body and their application to healthy volunteers does not show any side effects [9]. Because of that, a lot of *Acinetobacter baumannii* phages were isolated in recent years. Unfortunately, a majority of these phages are poorly characterized and especially, a lack of in-depth genome exploration poses a problem for their therapeutic use [10,11]. Thus suggesting that isolation and characterization of novel lytic bacteriophages is urgently needed. 

Here we fully characterize novel phage AGC01 (vB_AbaP_AGC01). Additionally, besides phenotypic and genomic characterization, we aimed to investigate the antimicrobial activity of AGC01 alone and in combination with antibiotics with the use of ex vivo and in vivo models. Enhancement of the phage activity by antibiotic addition was observed for e.g., *Escherichia coli*, *Burkholderia cepacia*, *Pseudomonas aeruginosa* [12,13]. Nevertheless, up to date only one *A. baumannii* infecting lytic phage KARL-1 was evaluated for antibiotic enhancement; result shows that MER augmented KARL-1 activity, but this study shows only in vitro-method based approach [11]. 

## 2. Results

### 2.1. Bacteriophage Isolation and Host Range

**Figure 1:**
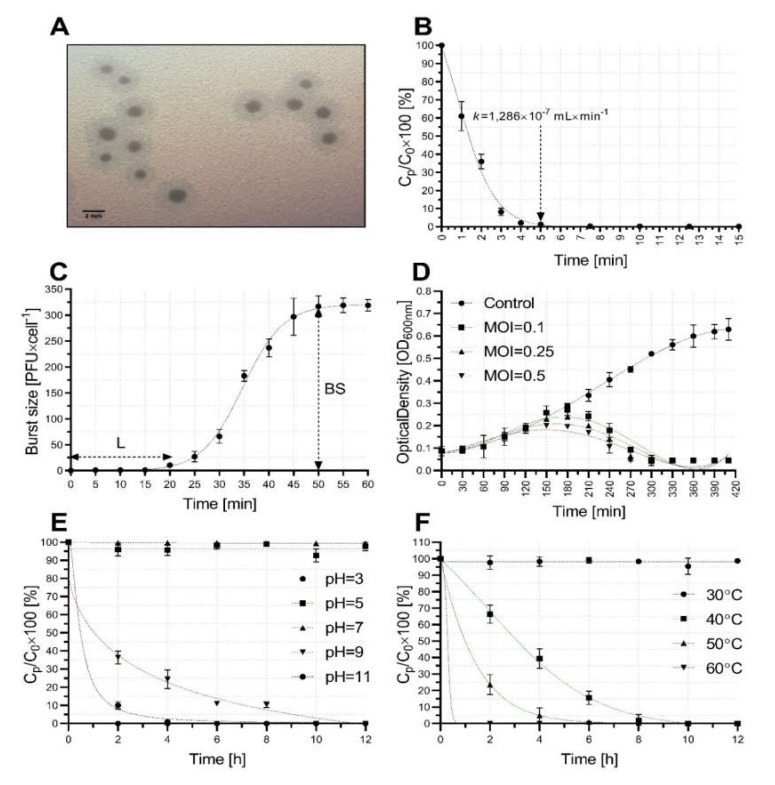
Characterization of bacteriophage vB_AbaP_AGC01 growth and stability. (**A**) Plaques formed by bacteriophage vb_AbaP_AGC01 after 18 h incubation at 37 °C. (**B**) Kinetics of the phage adsorption to host at a multiplicity of infection (MOI) of 0.1. (**C**) One-step growth curve indicating the latent period (L = 20 min) and burst size (BS = 317 PFU×cell^−1^). (**D**) Lytic activity of bacteriophage. (**E**) Stability of bacteriophages at different pH values. All experiments were technically repeated three times with triplicate biological replication. (**F**) Susceptibility of isolated bacteriophage to increases in temperature.

The isolated phage formed clear plaque surrounded by a halo zone (size: 1.6 ± 0.3 mm in diameter). Over prolonged incubation the plaque size and halo zone increased to 4.6 ± 0.4 mm (Figure 1A). The phage AGC01 was able to infect 50.27% (93 of 185) of the *A. baumannii* strains, including reference strains and clinical isolates (Table S1). Host range analysis showed that the AGC01 only infects *A. baumannii* and does not infect other species tested during this analysis (including *Escherichia coli*, *Klebsiella* spp., *Enterobacter* spp., and *Pseudomonas* spp. strains). 

### 2.2. Bacteriophage Growth and Stability Characterization

The virulence of AGC01 phage was initially characterized in terms of growth and stability under various conditions. First, an adsorption analysis showed that approximately 99% of virions adsorb to host within 5 min (Figure 1B). The latent period of AGC01 was found to be 20 min long and the burst size comprised approximately 317 ± 20 progeny phages per infected cell (Figure 1C). Finally, the ability of the phage to lyse *A. baumannii* cells was evaluated. Complete clearance of the bacterial optical density was observed in *A. baumannii* liquid culture and attributed to efficient lysis of cells by AGC01 at all the multiplicities of infection (MOIs) used (Figure 1D). The AGC01 was therefore found to possess strong and concentration-dependent lytic activity against the strain used. 

The resistance of phage AGC01 to pH and temperature changes was investigated to assess the stability of the phage under various physicochemical conditions. Phage AGC01 remains active at pH values ranging from 5 to 7, but loses activity at pH 3. When incubated at pH 9, within 2 h only 37% of virus particles remained active and able to infect the host, which further dropped to 10% active virus particles after exposure to pH 11 (Figure 1E). Analysis of thermal stability of bacteriophage showed that AGC01 retains activity at 30 °C throughout the duration of this analysis, while time-dependent decreases in phage activity were observed at temperatures 40 °C and 50 °C, and temperatures higher than 60 °C resulted in immediate loss of phage activity (Figure 1F). Additionally, storage of AGC01 phage stock at 4 °C resulted in loss of only 13% of active virions after 14 months.

### 2.3. Bacteriophage Comparative Genomics and Genome Analysis

**Figure 2:**
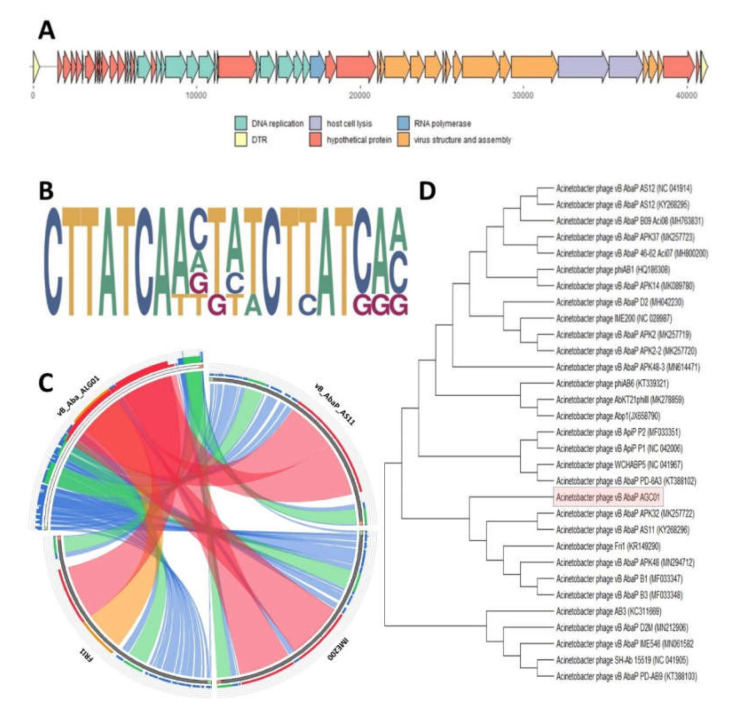
Genome organization, promoter structure, and comparative genomics of *Acinetobacter* phage vB_AbaP_AGC01. (**A**) Schematic presentation of the AGC01 genome organization. Arrows represent open reading frames (ORFs) identified in the genome and their transcriptional orientation. The coloring of genes represents the function of the genes: DNA replication (green), direct terminal repeat (DTR, yellow), virus structure and assembly (orange), host cell lysis (violet), hypothetical proteins (red). (**B**) Predicted putative phage promoter consensus of bacteriophage vb_AbaP_AGC01. (**C**) Circoletto visualization of the genome nucleotide sequence-structure similarity of the isolated phage with phages vB_AbaP_AS11 (acc. no. NC_041915.1), IME200 (acc. no. KT804908.2) and FRI1 (acc. no. KR149290.1). Ribbon colors correspond to similarity level (blue ≤ 0.25; green 0.26–0.5, orange 0.51–0.75, red > 0.75). (**D**) RNA polymerase-based phylogenetic tree of *Acinetobacter* infecting podoviruses based on RNA polymerase sequence.

The AGC01 phage genome (dsDNA, length: 41,231 bp) has a G+C content (39.50%) and a coding capacity (94.18%) similar to other *A. baumannii* infecting phages [14]. Additionally, this G+C content between 38.70% and 42.60% is similar to the G+C content of *A. baumannii* host strains [15]. The genome structure of bacteriophage vB_AbaP_AGC01 features high similarity to other podoviruses from the Friunavirus genus in a subfamily of *Autographivirinae*. The highest similarity to the AGC01 genome was observed for *Acinetobacter baumannii* phage vB_AbaP_APK92 (acc. no. MK257721.1), with query coverage of about 96% and identity of 94.71%. The sequence comparison between bacteriophages vB_AbaP_AS11, IME200, and FRI1 are presented in Figure 2C. RNA polymerase (RNAP)-based phylogenetic analysis confirmed the classification of the isolated bacteriophage as a Friunavirus genus member in a subfamily of *Autographivirinae*. The vB_AbaP_AGC01 phage was grouped in one clade together with Fri1 phage and vB_AbaP_AS11 (Figure 2D). Additionally, multiple genome alignments, showing conserved genomic sequence and rearrangements, of the selected *A. baumannii* infecting podoviruses from *Autographivirinae* are presented in Supplementary Figure S1.

In total, 55 open reading frames (ORFs) were identified in the AGC01 genome, and the functions of 32 genes (58 %) were assigned as shown in Figure 2A and Supplementary Table S2. The genome is organized into functional modules that contain genes involved in DNA replication, RNAP, virus structure, and assembly, as well as host cell lysis (Supplementary Table S2). Moreover, the AGC01 lysis cassette, which encodes holin and endolysin, is spaninless, which strongly suggests that AGC01 uses the canonical holin-endolysin lysis pathway similar to that described for the phage Petty lysis system [16]. The regulatory gene promoters were identified and the putative phage promoter consensus (based on 7 identified promoters) is presented in Figure 2B.

Importantly, a lack of integrases, as well as other proteins involved in lysogeny, host conversion and toxins, supports the growth kinetics data, suggesting that AGC01 possesses lytic properties and could be used for therapeutic purposes.

### 2.4. Activity of vB_AbaP_AGC01 on Biofilm, HIP-B, and G. Mellonella Larva Models

**Figure 3:**
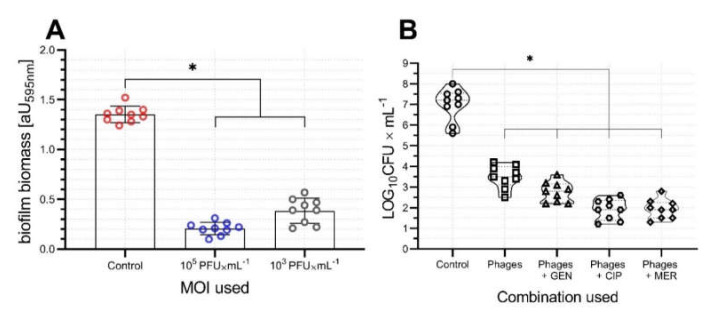
Activity of vB_AbaP_AGC01 with the use of the selected models. (**A**) Antibiofilm activity of isolated phage. (**B**) Antibacterial activity of AGC01 in human heat-inactivated plasma blood model alone and in combination with the antibiotics meropenem (MER), ciprofloxacin (CIP), and gentamicin (GEN). The asterisk (*) indicates statistically significant data (*p* < 0.05). All experiments were technically repeated three times with triplicate biological replication.

To evaluate bacteriophage activity and suitability as an antibacterial agent, we first analyzed the antibiofilm activity of AGC01. The biofilm biomass of infection-susceptible *A. baumannii* was reduced to 71.57% and 84.76% (*p* < 0.05) after incubation with AGC01 phage (Figure 3A). These data indicate that AGC01 possesses a high ability to reduce biofilm production.

To assess the ex vivo activity of AGC01 in blood, a heat-inactivated plasma blood model (HIP-B) was used. Phage AGC01 activity significantly reduced the MDRAB cell count when used alone and in combination with antibiotics in the HIP-B model (Figure 3B). The highest reduction of bacterial cell count was observed using phage in combination with CIP and MER, both of which resulted in an approximately 4 log reduction (*p* < 0.05, compared to phage used alone). The poorest efficiency was observed using the phage and GEN combination, where only a slight reduction of bacterial cell count was achieved which was not found to be significant (*p* = 0.0667). The use of the antibiotic alone does not significantly influence the bacterial count compared to control conditions (Supplementary Figure S2A).

**Figure 4:**
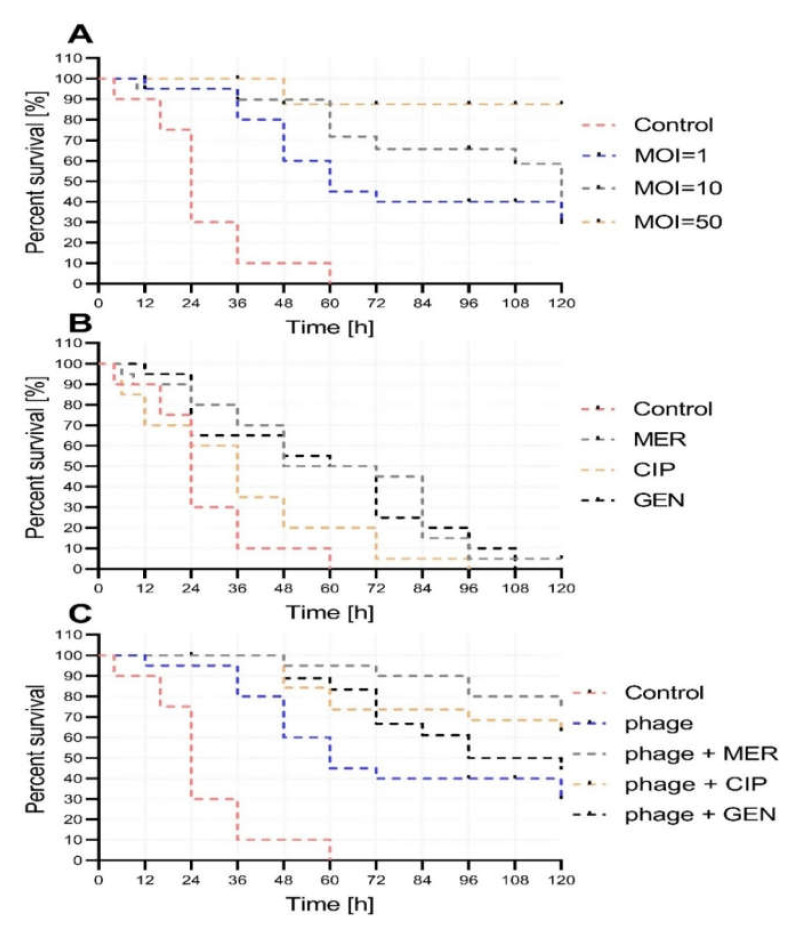
Survival of *G. mellonella* larvae. (**A**) Treatment of the *A. baumannii* (approx. 4 × 10^6^ CFU) infected larvae at different MOIs of vB_AbaP_AGC01. (**B**) Analysis of the influence of the antibiotics meropenem (MER), ciprofloxacin (CIP), and gentamicin (GEN) on the survival of *A. baumannii*-infected larvae. (**C**) The activity of antibiotics (MER, CIP, and GEN) in combination with AGC01 phage (MOI = 1) on survival of *A. baumannii* infected *G. mellonella* larvae. Survival of larvae treated with buffer, antibiotics, or phage alone was 100% (these lines were skipped for clarity). All experiments were technically repeated three times with triplicate biological replication.

Using an in vivo *G. mellonella* larva model, reduced mortality was observed in larvae infected with *A. baumannii* when phage treatment was introduced. The highest increase in survival rate was observed using bacteriophage treatment at an MOI of 50, while lower MOIs (10 and 1) also increased the survival rate of infected larvae (Figure 4A). All MOIs resulted in a significant increase in larva survival when compared to untreated control (*p* < 0.05).

Treatment with the antibiotics alone CIP, GEN, and MER did not notably improve survival of *A. baumannii* infected larvae, demonstrating the poor effectiveness of these antibiotics against MDRAB (Figure 4B). No statistically significant change in survival was observed for infected larvae treated with CIP (*p* = 0.08), MER, or GEN (*p* = 0.73). Treatment with MER resulted in a survival rate of 5% of larvae, while treatment with GER and CIP were not able to recover any larvae survival (all *p* < 0.05). Additionally, larvae lethality or melanization was not observed after antibiotics alone injection.

The combination of the antibiotics MER and CIP with phage treatment improved survival of *A. baumannii*-infected *G. mellonella* larvae compared to the single phage treatment, as well as relative to larvae treated with phage only (Figure 4C). The highest increase in survival rate (from 35% to 77%) was observed in infected larvae treated with a combination of phage (MOI = 10) and MER (*p* < 0.05). All combinations used had a significant increase of larva survival if compared to bacterial injected control (*p* < 0.05).

## 3. Discussion

Here, *Acinetobacter baumannii* infecting phage vB_AbaP_AGC01 was isolated, deeply characterized (phenotypically and genomically), and analyzed for antimicrobial activity in two infection models, namely HIP-B and *G. mellonella* larvae. Additionally, the influence of three different antibiotics (CIP, GEN, and MER) on phage activity in these models was determined. MER and CIP appeared to improve therapeutic outcomes of phage therapy in the in vivo model. The cumulative evidence supports the potential of vB_AbaP_AGC01 for therapeutic application alone or as a part of phage cocktails. Additionally, antibiotics (especially CIP and MER) improved therapeutic outcomes of phage therapy in in vivo model. However, to the best our knowledge this is the first study showing an antibiotic enhancement of the fully characterized lytic phage-based therapy against *A. baumannii* in human heat-inactivated plasma blood and *Galleria mellonella* model. The results suggest that the phages and antibiotics can act complementarily when administered together. 

The vB_AbaP_AGC01 was classified (based on genome sequence similarity) as a member of the Friunavirus genus from a subfamily of *Autographivirinae*. Isolated AGC01 phage possesses a broad lytic spectrum and a high target specificity, highlighting its potential as a tool for phage therapy of aforementioned infections or as a component of a phage cocktail against *A. baumannii* [17,18]. The genome of the AGC01 was characterized and annotated, which meets the requirements of deep genome analysis of phages candidates for therapeutic purposes [19]. Additionally, the lytic spectrum of around 50% suggests a broad host range of isolated phage. While, the host range of other Acinetobacter infecting phages varied between 2–68%, and in some reports, newly isolated phages infect only their propagation host [11,14,20]. Additionally, the host range of acinetobacter-infecting podoviruses mainly depended on pectate lyase depolymerase domains located on the tail fibers [16,21,22,23,24]. It can be presumed that isolation and characterization of naturally occurring *Acinetobacter* phages is important and puts novel insights into their biology. Additionally, they could be used as a source of capsule degrading enzymes that also poses antimicrobial properties [22]. Production of the depolymerases could be related to bacteriophage-mediated biofilm disruption. Isolated phage is able to reduce biofilm biomass at all concentrations, and this suggests that AGC01 exhibits antibiofilm properties. During the phage-antibiotic combination therapy, phage-mediated biofilm disruption increased antibiotic penetration and antibiofilm activity [25]. Limited penetration of antibiotics throughout the biofilm is contributed to increased biofilm resistance to antibiotics. 

Bacteriophages are very resilient to unfavorable environmental factors, such as temperature or pH changes [26,27]. In contrast, they are sensitive to divalent cation availability, which plays an essential role in the maintenance of the phage activity [28]. A decrease in the concentration of divalent metal ions, likely as a result of the EDTA chelation, was found to substantially restrict bacteriophage vB_AbaP_AGC01 activity when cations were not reconstructed (Supplementary Figure S2B). Similar findings were reported by Ma et al. (2018) [28], who observed that the lack of ion reconstruction decreased activity of bacteriophages infecting extraintestinal pathogenic *E. coli* strains in a HIP-B model. Additionally, phage antibacterial activity in HIP-B was suppressed by exogenous iron, magnesium, and calcium addition [28]. The present study illustrates the importance of divalent ions in determining phage activity in human blood and human blood-based sepsis models. Many other blood components, including hormones, lipids, carbohydrates, and metabolites, may also play a role in the determination of bacteriophage activity in blood. 

To the best of our knowledge, to date, there is no report describing the activity of *A. baumannii* infecting phages combined with antibiotics in a human blood-based model. The present study demonstrates that AGC01 in combination with CIP and MER significantly decreases the bacterial cell load in such a model. The enhanced activity of bacteriophages combined with different classes of antibiotics in liquid bacterial culture has been previously described. The *A. baumannii* infecting phage KARL-1 combined with therapeutic doses of colistin significantly increases the clearance of bacterial cells [11]. In addition, MER augmented KARL-1 activity at a MOI = 10^−7^, which is similar to the manner in which AGC01 and MER in combination exhibit a synergistic increase in bacterial clearance. Blasco et al. (2019) observed that the synergistic effect of a Ab105-2φΔCI lytic mutant combined with MER was maintained when ¼ MIC (minimal inhibitory concentration) and a MOI = 10 were used [29]. Unfortunately, no synergistic effect was observed using KARL-1 in combination with CIP [11]. The combination of AGC01 with CIP resulted in similar enhancement of phage activity as was seen within combination with MER. The strain dependence of the PAS effect has been previously described by Pletezer et al. (2018) where the PAS effect was observed on 86.6% of *Pseudomonas aeruginosa* strains analyzed [30]. These variations observed suggest that the PAS effect may be a strain and/or phage dependent. Additionally, the medium or model used may also play an important role in this phenomenon. The PAS effect occurrence should be analyzed with the use of the in vivo and ex vivo models for each bacterial strain and phage respectively before the use of in vivo models. 

The in vitro conditions used hitherto are not representative of animal infection models. Ideally, therapeutic evaluation of bacteriophages as candidates for phage therapy should include ex vivo and in vivo models. This motivated the evaluation of the antimicrobial activity of AGC01 and antibiotics in a greater wax moth larva (*G. mellonella*) model. *G. mellonella* was proposed as an alternative for the evaluation of the activity and toxicity of new antimicrobial agents [31]. Previous studies have shown the utility of this model in the assessment of phage therapy against *Bordetella bronchiseptica*, *Cronobacter sakazakii*, *P. aeruginosa*, and *Burkholderia cepacia* [19,32,33,34,35]. This model was also used for assessment of the phage therapy treatment against *A. baumannii* [35]. High MOI (100) single-dose phage Bφ-R2096 therapy increased the survival rates of larvae by approximately 50%, 24 h after *A. baumannii* infection [35]. We demonstrated that AGC01 treatment increases the survival rate of larvae from 30% to 90% at a MOI of 50, as well as observing a statistically significant increase in the survival rate of *G. mellonella* larvae treated with AGC01 at lower MOIs (1 and 10). The combination of phage with all used antibiotics was significantly more active than antibiotics used alone, and conversely, the combination of phage with CIP and MER statistically increased the effectiveness of phage therapy. MER was previously combined with bacteriophage KS12 infecting *Burkholderia cepacia* and the combined treatment resulted in a 20% decrease in mortality after 48 h and a 56% decrease after 72 h (when bacteria infected control group reached 100% mortality) [13]. Also, MER augmented KARL-1 activity in vitro-method-based approach but this study lacks in vivo analysis [11]. Importantly, after the injection of phages, antibiotics, or combinations of these agents, the melanization marker of the larva humoral response was not detected [36], which suggests that combined therapy based on bacteriophages and antibiotics may be both highly effective and safe. Increasements of the activity of phages combined with antibiotics might be promoted by bacterial cell stress response, which might promote phage replication inside of cell [11]. Synergistic action between antibiotics and bacteriophages might be extremely beneficial for infection treatment outcomes. Unfortunately, the actual knowledge of this phenomenon is limited and mostly speculative [37].

In conclusion, these results suggest that combined therapy poses the highest effectiveness, results should be considered with care. Especially, observed PAS effect is often strain-specific and results obtained with the use of the invertebrate models of *A. baumannii* infection need additional mammalian infection models for confirmation. Further investigations are necessary to understand and precise molecular mode of action of the phages-antibiotics combinations. 

## 4. Materials and Methods 

### 4.1. Identification, Storage, and Growth of Bacterial Strains

*Acinetobacter baumannii* (ATCC^®^16909^™^) was obtained from ATCC (Manassas, VA, USA). In addition, 185 MDRAB strains isolated from clinical specimens were accessed at the Pomeranian Medical University in Szczecin (Szczecin, Poland). Strains were characterized using a VITEK^®^2 Compact Bacterial Identification System (bioMérieux, Warsaw, Poland) and validated by MALDI-TOF mass spectrometry. Antibiotic susceptibility was assessed according to CLSI guidance. Bacterial strains were stored at −80 °C before use. Bacterial strains were grown on blood agar (bioMérieux, Warsaw, Poland) for 24 h at 37 °C to revitalize prior to further application.

### 4.2. Bacteriophage Isolation, Purification, Propagation, and Host Range

Bacteriophage was isolated from fishery pond sample (collected in Stargard, West Pomeranian Region, Poland) using *A. baumannii* ATCC^®^16909^™^ as a host with the use of the standard enrichment technique [38]. After that, bacteriophage was triple time propagated from a single plaque for strain purification. Following the guidelines of Adriaenssens and Brister [39], isolated bacteriophage was designated vB_AbaP_AGC01 (also referred as AGC01). A high titer phage lysate was prepared by overnight propagation in a log-phase bacterial culture with shaking (180 rpm) at 37 °C. The phage lysate was subsequently centrifuged at 2000× *g* for 10 min at 4 °C, and supernatant was filtered through a 0.2 µm sterile filter (Sartorius Stedim Biotech, France). 

The phage titer, and host range was determined by means of a double agar overlay or double layer agar (DLA) plaque assay as previously described [40]. A total of 185 clinical MDRAB strains, isolated from nosocomial infections in West Pomeranian and Lubusz regions of Poland, were used to assess the host range of the isolated bacteriophage. Host range analysis based on the efficacy of plating (EOP), allowed the exclusion of false-positive results [41]. The EOP was calculated according to Equation (1).

(1) $$\mathrm{EOP}=\frac{\mathrm{average}\;\mathrm{PFU}\;\mathrm{on}\;\mathrm{target}\;\mathrm{bacteria}}{\mathrm{average}\;\mathrm{PFU}\;\mathrm{on}\;\mathrm{host}\;\mathrm{bacteria}}$$

### 4.3. Bacteriophage Characterization

Adsorption assay was performed as previously described [19]. The number of adsorbed phages was determined as a percent decrease in PFUs. The adsorption constants were calculated as previously recommended [42] following Equation (2).

(2) $$k=\frac{2.3}{\mathrm{bacterial}\;\mathrm{initial}\;\mathrm{count}\;\left[\mathrm{CFU}\times {\mathrm{mL}}^{-1}\right]\;\times \;\mathrm{time}\;\left[\min\right]}{log}_{10}\left(\frac{\mathrm{phage}\;\mathrm{initial}\;\mathrm{counts}\;\left[\mathrm{PFU}\;\times \;{\mathrm{mL}}^{-1}\right]}{\mathrm{phage}\;\mathrm{count}\;\mathrm{at}\;\mathrm{time}\;\mathrm{point}\;\left[\mathrm{PFU}\;\times \;{\mathrm{mL}}^{-1}\right]}\right)$$

One-step growth, whereby isolated bacteriophages infect *A. baumannii* cells in a single cycle only, was performed, as described by Turner et al. [14] with minor modifications. Briefly, bacteriophages at a MOI of 0.05 were added to the bacterial culture and allowed to adsorb for 5 min. Afterward, the sample was centrifuged at 13,000× *g*, the supernatant was removed and the pellet was resuspended in equal to the initial volume of LB broth (A&A Biotechnology, Poland). The sample was diluted to 10^−7^, incubated and titrated by triplicate overlay plaque assay to determine the single burst.

Bacteriophage lysis of liquid culture was evaluated as follows: Fresh LB medium supplemented with 10 mM CaCl_2_ (Sigma-Aldrich, Darmstadt, Germany) and MgCl_2_ (Sigma-Aldrich, Darmstadt, Germany) medium was inoculated using overnight *A. baumannii* cultures and was incubated shaking at 180 rpm at 37 °C to reach an optical density at 600 nm (OD_600nm_) of 0.4. At that point, bacterial cultures were treated with bacteriophage at MOIs of 0.1, 0.25, 0.5. Samples were collected every 30 min after bacteriophage addition and the OD_600nm_ was measured using an En-Vision 2105 multimode spectrophotometric plate reader (Perkin-Elmer, USA). Growth medium was used as the blank sample and bacterial culture with phage-free buffer and antibiotics added was used as growth control. The experiment was technically repeated three times, conducted in triplicate.

The thermal and pH stability of the AGC01 was conducted as previously described [43].

Phage vB_AbaP_AGC01 DNA was isolated with the use of the Genomic Mini AX Phage kit (A&A Biotechnology, Poland). The complete genome sequence of AGC01 was determined in the DNA Sequencing and Oligonucleotide Synthesis Laboratory (oligo.pl) at the Institute of Biochemistry and Biophysics, Polish Academy of Sciences (Warsaw, Poland). The complete and fully annotated genome sequence of the *A. baumannii* phage vB_AbaP_AGC01 is available from the GenBank database (acc. no. MT263719).

Prediction of open reading frames (ORFs) and genome annotations were performed with the use of Geneious Prime 2019.2.1 software (https://www.geneious.com). Genome annotations were verified by comparison of the related protein sequence with the use of blast software (blastp.ncbi.nlm.gov.pl). RNAP-based phylogeny was prepared with the use of MEGA X software [44]. Phage regulatory elements were scanned with the use of PHIRE software [45] and visualized using WebLogo [46]. The sequences of isolated bacteriophages were aligned and similarity was verified with the use of Circoletto software [47]. The bacteriophages vB_AbaP_AS11 (acc. no. NC_041915.1), IME200 (acc. no. KT804908.2), and FRI1 (acc. no. KR149290.1) were used for generation of sequence similarity graphs.

### 4.4. Analysis of Bacterial Growth and Bacteriophage Activity in Blood Model

All blood sample donors gave their informed consent for inclusion before they participated in the study. The study was conducted in accordance with the Declaration of Helsinki and the protocol was approved by the Local Ethics Committee (KB-0012/33/19).

Whole human blood was collected from healthy volunteers by means of a sterile syringe containing EDTA (S-Monovette®, Sarstedt, Nümbrecht, Germany). Analysis of bacteriophage and antibiotic synergistic activity was performed in heat-inactivated plasma blood (HIP-B) according to Ma et al. (2018) [28] with minor modifications. HIP-B was prepared by centrifugation of the whole blood samples at 2000× *g* for 10 min and the plasma was heat-inactivated at 56 °C for 1 h. The pelleted erythrocytes and leukocytes were rinsed with sterile PBS buffer preheated to 37 °C. The heat-inactivated plasma was added to other blood components for blood reconstruction. For the reconstruction, CaCl_2_, FeSO_4_, or MgCl_2_ (all purchased from Sigma-Aldrich, Darmstadt, Germany) ions were added to a final concentration of 5 mM. Bacteriophage activity was measured in blood reconstructed with ions and in non-reconstructed blood. 

To assess the growth rate of *A. baumannii* clinical strains in HIP-B, the blood sample was inoculated with 50 µL of *A. baumannii* overnight culture and incubated for 24 h at 37 °C. Afterward, serial dilutions were prepared, plated on trypticase soy agar (TSA) (A&A Biotechnology, Poland), and incubated for 24 h at 37 °C. Bacteriophage activity was assessed by inoculation of preheated HIP-B with *A. baumannii* at a final concentration of 1.5 × 10^5^ CFU × mL^−1^ and adding vB_AbaP_AGC01 at a MOI = 10 before incubation at 37 °C. To assess the influence of antibiotics on phage activity, MER (20 mg × L^−1^) (Sigma-Aldrich, Darmstadt, Germany), CIP (10 mg × L^−1^) (Sigma-Aldrich, Darmstadt, Germany), and GEN (8 mg × L^−1^) (Sigma-Aldrich, Darmstadt, Germany) were added to cultures as indicated. Samples were collected after 12 h of incubation at 37 °C. Serial dilutions were prepared of each experimental culture in PBS and these were plated on a TSA plate and incubated for 18–24 h at 37 °C. Bacterial colonies that grow in the presence of bacteriophage and bacteriophage/antibiotic combination were analyzed for phage sensitivity using the spot-test methodology.

### 4.5. In Vivo Synergy in the Galleria Mellonella Model

Only larvae exhibiting a uniform cream color and weighing 300 mg (slight variations were used to calculate antibiotic dosage) were used for experiments. The in vivo test was performed as described by Szymczak et al. (2020) [19] with minor modifications. Briefly, *G. mellonella* larvae were infected with approximately 4 × 10^6^ CFU of *A. baumannii* into the larval hemolymph behind the last proleg. After 20 min, the infected larvae were treated with the bacteriophage suspension at MOI ≈ 1, which was injected on the opposite side to the bacterial injection. Where antibiotics were used, these were premixed into the phage solution to the following final concentrations: 20 mg × kg^−1^ MER, 10 mg × kg^−1^ CIP, or 8 mg × kg^−1^ GEN. Doses were based on clinically relevant doses applied to human patients. Buffer, phage suspension, and antibiotic suspension controls were prepared when assessing bacteriophage toxicity. Larvae were incubated at 37 °C and examined every 12 h. The dark larvae that did not respond to physical contact were marked deceased.

### 4.6. Statistical Analysis

Data were analyzed using one-way analysis of variance (ANOVA). *p* values lower than 0.05 were considered statistically significant. Kaplan-Meier survival test was used for analysis of *G. mellonella* survival with the Mantel-Cox test. All statistical analyses were carried out using GraphPad Prism 7.04 software (Graph Pad Software, San Diego, CA, USA).

## Supplementary Materials

Supplementary materials can be found at https://www.mdpi.com/1422-0067/21/12/4390/s1.

## Abbreviations

ANOVA	Analysis of variance
CFU	Colony-forming unit
CIP	Ciprofloxacin
CLSI	Clinical and Laboratory Standard Institute
DLA	Double layer agar
GEN	Gentamicin
*k*	Adsorption constant
LB	Luria Bertani broth
MALDI	Matrix-assisted laser desorption/ionization
MDRAB	Multidrug resistant *Acinetobacter baumannii*
MER	Meropenem
MOI	Multiplicity of infection
NCBI	National Center for Biotechnology Information
OD	Optical density
ORF	Open reading frame
PAS	Phage antibiotic synergy
PBS	Phosphate buffered saline
PFU	Plaque forming units
RNAP	RNA polymerase
SD	Standard deviation
TOF	Time-of-flight
TSA	Trypticase soy agar
