# Supplementary material for: Antibiotics Act with vB_AbaP_AGC01 Phage against Acinetobacter baumannii in Human Heat-Inactivated Plasma Blood and Galleria mellonella Models
Source: Int J Mol Sci. 2020 Jun 19;21(12):4390. doi: 10.3390/ijms21124390 (PMC7352404; doi:10.3390/ijms21124390)
Supplement: Supplementary file 1 [file ijms-21-04390-s001.pdf]

International Journal of *Molecular Sciences*

MDPI

Supplementary materials to

Antibiotics act in concert with novel Fri1-like bacteriophage vB_AbaP_AGC01 against multidrug resistant *Acinetobacter baumannii* in human heat inhibited plasma blood and *Galleria mellonella* models

Bartłomiej Grygorcewicz [1*], Marta Roszak [1], Piotr Golec [2], Daria Śleboda-Taront [1], Natalia Łubowska [3], Martyna Górska [4], Joanna Jursa-Kulesza [5], Rafał Rakoczy [6], Bartosz Wojciuk [7], and Barbara Dołęgowska [1]

[1] Department of Laboratory Medicine, Chair of Microbiology, Immunology and Laboratory Medicine, Pomeranian Medical University in Szczecin, Powstańców Wielkopolskich 72, 70-111 Szczecin, Poland; marta.roszak@pum.edu.pl (MR); daria.sleboda@pum.edu.pl (D.Ś-T.); barbara.dolegowska@pum.edu.pl (B.D.).

[2] Department of Molecular Virology, Institute of Microbiology, Faculty of Biology, University of Warsaw, Miecznikowa 1, 02-096 Warsaw, Poland; pgolec@biol.uw.edu.pl (P.G.).

[3] Department of Medical Microbiology, Faculty of Medicine, Medical University of Gdansk; Marii Skłodowskiej-Curie 3a, 80-210 Gdańsk, Poland; nlubowska@gumed.edu.pl

[4] Department of General Pathology, Pomeranian Medical University in Szczecin, Powstańców Wielkopolskich 72, 70-111 Szczecin, Poland;

[6] Department of Medical Microbiology, Chair of Microbiology, Immunology and Laboratory Medicine, Pomeranian Medical University in Szczecin, Powstańców Wielkopolskich 72, 70-111 Szczecin, Poland; asiaju@pum.edu.pl (J.J.-K.).

[7] Department of Diagnostic Immunology, Chair of Microbiology, Immunology and Laboratory Medicine, Pomeranian Medical University in Szczecin, Powstańców Wielkopolskich 72, 70-111 Szczecin, Poland; bartosz.wojciuk@pum.edu.pl (B.W.).

- Correspondence: bartlomiej.grygorcewicz@pum.edu.pl, b.grygorcewicz@gmail.com ;

Table S1. Host range of the vB_AbaP_AGC01 phage. EOP – efficiency of plating; BI -Blood infection; RTI – Respiratory tract infection; STI – Skin and soft tissue infection, UTI – Urinary tract infection.

No.	Internal code	Species	Source of isolation	EOP
**1**	G60/1	*Acinetobacter baumannii*	BI	0.79
**2**	G49/1	*Acinetobacter baumannii*	BI	0
**3**	G64/1	*Acinetobacter baumannii*	BI	0
**4**	G61/1	*Acinetobacter baumannii*	BI	0.83
**5**	G43/2	*Acinetobacter baumannii*	BI	0.97
**6**	G48/2	*Acinetobacter baumannii*	BI	0.83
**7**	G56/2	*Acinetobacter baumannii*	BI	1.2
**8**	G38/1	*Acinetobacter baumannii*	BI	0
**9**	G76/2	*Acinetobacter baumannii*	BI	0
**10**	G6/2	*Acinetobacter baumannii*	BI	1.02
**11**	G45/2	*Acinetobacter baumannii*	BI	1
**12**	G56/1	*Acinetobacter baumannii*	BI	0.6
**13**	G32/1	*Acinetobacter baumannii*	BI	0
**14**	G54/2	*Acinetobacter baumannii*	BI	0
**15**	G39/1	*Acinetobacter baumannii*	BI	0
**16**	G63/1	*Acinetobacter baumannii*	BI	0.46
**17**	G4/2	*Acinetobacter baumannii*	BI	0.92
**18**	G80/1	*Acinetobacter baumannii*	BI	0
**19**	G9/3	*Acinetobacter baumannii*	BI	0
**20**	G5/3	*Acinetobacter baumannii*	BI	0
**21**	G15/2	*Acinetobacter baumannii*	RTI	0
**22**	G16/2	*Acinetobacter baumannii*	RTI	0
**23**	G41/1	*Acinetobacter baumannii*	RTI	0
**24**	G21/2	*Acinetobacter baumannii*	RTI	0
**25**	G24/1	*Acinetobacter baumannii*	RTI	0
**26**	G25/1	*Acinetobacter baumannii*	RTI	0.98
**27**	G59/1	*Acinetobacter baumannii*	RTI	0.74
**28**	G40/2	*Acinetobacter baumannii*	RTI	0
**29**	G37/2	*Acinetobacter baumannii*	RTI	0
**30**	G27/1	*Acinetobacter baumannii*	RTI	0
**31**	G42/1	*Acinetobacter baumannii*	RTI	0
**32**	G26/1	*Acinetobacter baumannii*	RTI	0.5
**33**	G2/2	*Acinetobacter baumannii*	RTI	0
**34**	G71/2	*Acinetobacter baumannii*	RTI	0.61
**35**	G1/1	*Acinetobacter baumannii*	RTI	0.73
**36**	G11/2	*Acinetobacter baumannii*	RTI	0
**37**	G22/2	*Acinetobacter baumannii*	RTI	0
**38**	G77/1	*Acinetobacter baumannii*	RTI	0
**39**	G74/1	*Acinetobacter baumannii*	RTI	1.2
**40**	G12/2	*Acinetobacter baumannii*	RTI	0
**41**	G57/1	*Acinetobacter baumannii*	RTI	0.23
**42**	G58/1	*Acinetobacter baumannii*	RTI	0.47
**43**	G35/2	*Acinetobacter baumannii*	RTI	0.84
**44**	G55/2	*Acinetobacter baumannii*	RTI	0.73
**45**	G64/2	*Acinetobacter baumannii*	RTI	0

**46**	G10/2	*Acinetobacter baumannii*	RTI	0.54
**47**	G54/1	*Acinetobacter baumannii*	RTI	1.41
**48**	G36/2	*Acinetobacter baumannii*	RTI	1
**49**	G60/2	*Acinetobacter baumannii*	RTI	1.12
**50**	G59/2	*Acinetobacter baumannii*	RTI	0.92
**51**	G34/2	*Acinetobacter baumannii*	RTI	0
**52**	G70/2	*Acinetobacter baumannii*	RTI	0
**53**	G4/1	*Acinetobacter baumannii*	RTI	0
**54**	G23/2	*Acinetobacter baumannii*	RTI	0.96
**55**	G20/1	*Acinetobacter baumannii*	RTI	0.57
**56**	G20/2	*Acinetobacter baumannii*	RTI	0.29
**57**	G62/2	*Acinetobacter baumannii*	RTI	0.66
**58**	G32/2	*Acinetobacter baumannii*	RTI	0.39
**59**	G1/2	*Acinetobacter baumannii*	RTI	0
**60**	G63/2	*Acinetobacter baumannii*	RTI	0
**61**	G3/1	*Acinetobacter baumannii*	RTI	0
**62**	G36/1	*Acinetobacter baumannii*	RTI	0.78
**63**	G58/2	*Acinetobacter baumannii*	RTI	0
**64**	G9/1	*Acinetobacter baumannii*	RTI	0
**65**	G75/2	*Acinetobacter baumannii*	RTI	0
**66**	G5/2	*Acinetobacter baumannii*	RTI	0
**67**	G29/1	*Acinetobacter baumannii*	RTI	0
**68**	G78/2	*Acinetobacter baumannii*	RTI	0.49
**69**	G3/2	*Acinetobacter baumannii*	RTI	0.84
**70**	G74/2	*Acinetobacter baumannii*	RTI	0.76
**71**	G31/2	*Acinetobacter baumannii*	RTI	0
**72**	G46/2	*Acinetobacter baumannii*	RTI	0
**73**	G44/2	*Acinetobacter baumannii*	RTI	0
**74**	G51/2	*Acinetobacter baumannii*	RTI	0
**75**	G33/2	*Acinetobacter baumannii*	RTI	1.12
**76**	G2/1	*Acinetobacter baumannii*	RTI	0
**77**	G30/1	*Acinetobacter baumannii*	RTI	0
**78**	G73/2	*Acinetobacter baumannii*	RTI	0
**79**	G31/1	*Acinetobacter baumannii*	RTI	0
**80**	G5/1	*Acinetobacter baumannii*	RTI	0
**81**	G57/2	*Acinetobacter baumannii*	RTI	0.82
**82**	G62/1	*Acinetobacter baumannii*	RTI	0.46
**83**	G25/2	*Acinetobacter baumannii*	RTI	0
**84**	G61/2	*Acinetobacter baumannii*	RTI	0
**85**	G19/2	*Acinetobacter baumannii*	RTI	0
**86**	G16/1	*Acinetobacter baumannii*	RTI	0
**87**	G55/1	*Acinetobacter baumannii*	RTI	0.56
**88**	G79/1	*Acinetobacter baumannii*	RTI	0
**89**	G35/1	*Acinetobacter baumannii*	RTI	0.17
**90**	G17/1	*Acinetobacter baumannii*	RTI	0.93
**91**	G30/2	*Acinetobacter baumannii*	RTI	0
**92**	G50/1	*Acinetobacter baumannii*	RTI	0
**93**	G6/1	*Acinetobacter baumannii*	RTI	0.9
**94**	G81/1	*Acinetobacter baumannii*	RTI	0.83
**95**	G9/2	*Acinetobacter baumannii*	RTI	0.58

**96**	G7/3	*Acinetobacter baumannii*	RTI	0
**97**	G11/3	*Acinetobacter baumannii*	RTI	0
**98**	G93/01	*Acinetobacter baumannii*	RTI	0.93
**99**	G14/2	*Acinetobacter baumannii*	STI	0.34
**100**	G48/1	*Acinetobacter baumannii*	STI	0.29
**101**	G34/1	*Acinetobacter baumannii*	STI	0
**102**	G72/1	*Acinetobacter baumannii*	STI	0.92
**103**	G77/2	*Acinetobacter baumannii*	STI	0
**104**	G23/1	*Acinetobacter baumannii*	STI	0
**105**	G14/1	*Acinetobacter baumannii*	STI	0
**106**	G76/1	*Acinetobacter baumannii*	STI	0.62
**107**	G80/2	*Acinetobacter baumannii*	STI	0.21
**108**	G47/2	*Acinetobacter baumannii*	STI	0.81
**109**	G53/2	*Acinetobacter baumannii*	STI	0
**110**	G42/2	*Acinetobacter baumannii*	STI	1.13
**111**	G45/1	*Acinetobacter baumannii*	STI	1
**112**	G28/2	*Acinetobacter baumannii*	STI	0.67
**113**	G51/1	*Acinetobacter baumannii*	STI	0
**114**	G44/1	*Acinetobacter baumannii*	STI	0
**115**	G29/2	*Acinetobacter baumannii*	STI	0
**116**	G53/1	*Acinetobacter baumannii*	STI	0.64
**117**	G43/1	*Acinetobacter baumannii*	STI	0
**118**	G12/1	*Acinetobacter baumannii*	STI	0.76
**119**	G24/2	*Acinetobacter baumannii*	STI	0.39
**120**	G41/2	*Acinetobacter baumannii*	STI	0
**121**	G10/1	*Acinetobacter baumannii*	STI	0.59
**122**	G37/1	*Acinetobacter baumannii*	STI	0.34
**123**	G22/1	*Acinetobacter baumannii*	STI	0.22
**124**	G33/1	*Acinetobacter baumannii*	STI	0
**125**	G8/1	*Acinetobacter baumannii*	STI	1.34
**126**	G18/1	*Acinetobacter baumannii*	STI	0
**127**	G11/1	*Acinetobacter baumannii*	STI	0
**128**	G13/2	*Acinetobacter baumannii*	UTI	1
**129**	G67/2	*Acinetobacter baumannii*	UTI	0.59
**130**	G47/1	*Acinetobacter baumannii*	UTI	0.92
**131**	G65/1	*Acinetobacter baumannii*	UTI	0.64
**132**	G18/2	*Acinetobacter baumannii*	UTI	0
**133**	G75/1	*Acinetobacter baumannii*	UTI	0.53
**134**	G46/1	*Acinetobacter baumannii*	UTI	0
**135**	G17/2	*Acinetobacter baumannii*	UTI	0.48
**136**	G13/1	*Acinetobacter baumannii*	UTI	0
**137**	G15/1	*Acinetobacter baumannii*	UTI	0.85
**138**	G78/1	*Acinetobacter baumannii*	UTI	0
**139**	G73/1	*Acinetobacter baumannii*	UTI	0.79
**140**	G65/2	*Acinetobacter baumannii*	UTI	0.93
**141**	G38/2	*Acinetobacter baumannii*	UTI	1.2
**142**	G79/2	*Acinetobacter baumannii*	UTI	0.21
**143**	G71/1	*Acinetobacter baumannii*	UTI	0
**144**	G7/2	*Acinetobacter baumannii*	UTI	0
**145**	G70/1	*Acinetobacter baumannii*	UTI	0

**146**	G66/2	*Acinetobacter baumannii*	UTI	0.89
**147**	G7/1	*Acinetobacter baumannii*	UTI	0.71
**148**	G68/1	*Acinetobacter baumannii*	UTI	1.32
**149**	G66/1	*Acinetobacter baumannii*	UTI	0
**150**	G50/2	*Acinetobacter baumannii*	UTI	0.49
**151**	G52/1	*Acinetobacter baumannii*	UTI	0
**152**	G8/2	*Acinetobacter baumannii*	UTI	0
**153**	G49/2	*Acinetobacter baumannii*	UTI	0.83
**154**	G68/2	*Acinetobacter baumannii*	UTI	0.24
**155**	G69/2	*Acinetobacter baumannii*	UTI	00
**156**	G40/1	*Acinetobacter baumannii*	UTI	0
**157**	G72/2	*Acinetobacter baumannii*	UTI	0
**158**	G27/2	*Acinetobacter baumannii*	UTI	0.9
**159**	G19/1	*Acinetobacter baumannii*	UTI	0.34
**160**	G69/1	*Acinetobacter baumannii*	UTI	0
**161**	G21/1	*Acinetobacter baumannii*	UTI	0
**162**	G52/2	*Acinetobacter baumannii*	UTI	0
**163**	Ab1/113	*Acinetobacter baumannii*	UTI	1.2
**164**	Ab1/119	*Acinetobacter baumannii*	UTI	1.1
**165**	Ab1/123	*Acinetobacter baumannii*	UTI	0.46
**166**	Ab2/01	*Acinetobacter baumannii*	UTI	0.32
**167**	Ab2/02	*Acinetobacter baumannii*	UTI	0
**168**	Ab2/19	*Acinetobacter baumannii*	UTI	0.93
**169**	Ab2/87	*Acinetobacter baumannii*	UTI	0
**170**	Ab3/1	*Acinetobacter baumannii*	UTI	0
**171**	Ab4/12	*Acinetobacter baumannii*	UTI	0
**172**	Ab5/02	*Acinetobacter baumannii*	UTI	0
**173**	Ab5/16	*Acinetobacter baumannii*	UTI	0.12
**174**	Ab5/19	*Acinetobacter baumannii*	UTI	1.32
**175**	Ab5/23	*Acinetobacter baumannii*	UTI	0
**176**	Ab5/19	*Acinetobacter baumannii*	UTI	0.39
**177**	Ab5/22	*Acinetobacter baumannii*	UTI	0.66
**178**	Ab6/11	*Acinetobacter baumannii*	UTI	0
**179**	Ab6/12	*Acinetobacter baumannii*	UTI	0
**180**	Ab6/13	*Acinetobacter baumannii*	UTI	0.79
**181**	Ab6/14	*Acinetobacter baumannii*	UTI	0.39
**182**	Ab6/15	*Acinetobacter baumannii*	UTI	0.63
**183**	Ab6/16	*Acinetobacter baumannii*	UTI	0
**184**	ATCC®19606™	*Acinetobacter baumannii*	UTI	1
**185**	ATCC®17978™	*Acinetobacter baumannii*	UTI	0.43

Table S2. Predicted function of the open reading frames defined within the functional module.

Functional module	Predicted function (ORF)
DNA replication	DNA binding protein (ORFs: 14, 16, 22, 51)<br>DNA primase/helicase (ORFs: 17, 19)<br>DNA/RNA binding protein (ORF20)<br>DNA helicase (ORF21)<br>ATP-dependent DNA ligase (ORF23)<br>DNA polymerase I (ORF24),<br>deoxynucleoside monophosphate kinase (ORF27)<br>5′-3′ exonuclease (ORF28)<br>tRNA nucleotidyltransferase (ORF29)<br>DNA endonuclease VII (ORF30)<br>phosphoestherase with HTH domain (ORF31)<br>dNMP kinase (ORF32)
RNA polymerase (RNAP)	DNA-directed RNA polymerase (ORF33)
Virus structure and assembly	head-to-tail connector protein (ORF36)<br>scaffolding protein (ORF37)<br>major capsid protein (ORF38)<br>tail tubular protein A (ORF39)<br>tail needle protein (ORF40)<br>tail tubular protein B (ORF41)<br>tail tubular protein C (ORF42)<br>internal virion protein A (ORF43)<br>internal virion protein A (ORF44)<br>DNA ejectosome component (ORF45)<br>tail fiber protein (ORF46)<br>DNA maturase A (ORF49)<br>DNA maturase B (ORF50)
Host cell lysis	holin (ORF47)<br>endolysin (ORF48)

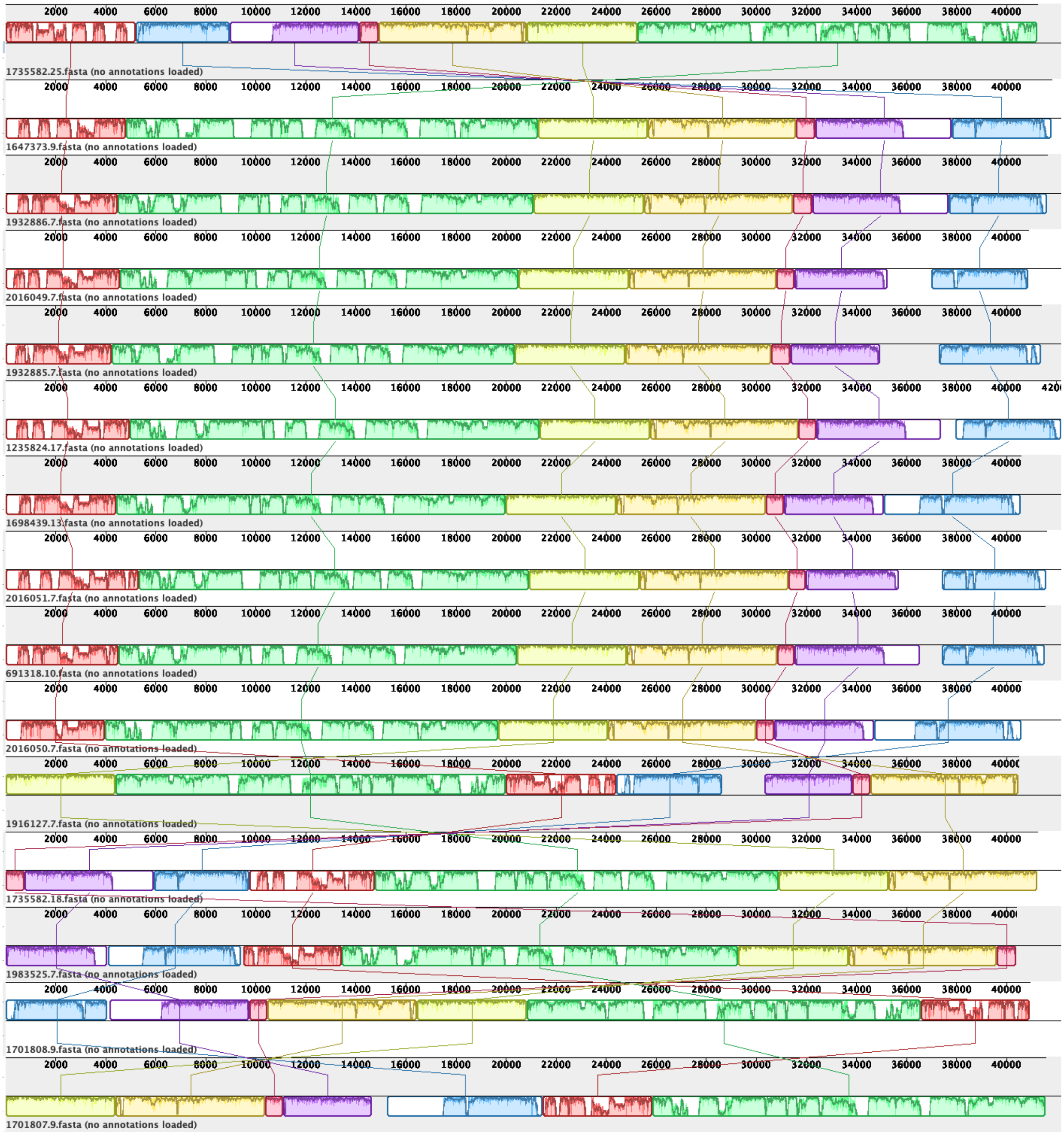

Figure S1. Mauve Colored boxes indicate homologous DNA regions between genomes without genomic rearrangements. Genomic similarity is represented by the height of the bars, which corresponds to the average level of conservation in that region of the genome sequence. Completely white regions represent fragments that were not aligned or contained sequence elements specific to a particular genome. Data were analyzed with PATRIC 3.6.3. [1] (1735582.25 – Acinetobacter phage vB_AbaP_AGC01; 1235824.17 - Acinetobacter phage Abp1; 1647373.9 - Acinetobacter phage Fri1; 1698439.13 - Acinetobacter phage phiAB6; 1701807.9 - Acinetobacter phage vB_AbaP_PD-6A3; 1701808.9 - Acinetobacter phage vB_AbaP_PD-AB9; 1735582.18 - Acinetobacter phage vB_AbaP_IME200; 1916127.7 - Acinetobacter phage SH-Ab 15519; 1932885.7 - Acinetobacter phage vB_AbaP_AS12; 1932886.7 - Acinetobacter phage vB_AbaP_AS11; 1983525.7 - Acinetobacter phage WCHABP5; 2016049.7 - Acinetobacter phage vB_AbaP_B1; 2016050.7 - Acinetobacter phage vB_AbaP_B3; 2016051.7 - Acinetobacter phage vB_AbaP_B5; 691318.10 - Acinetobacter phage phiAB1).

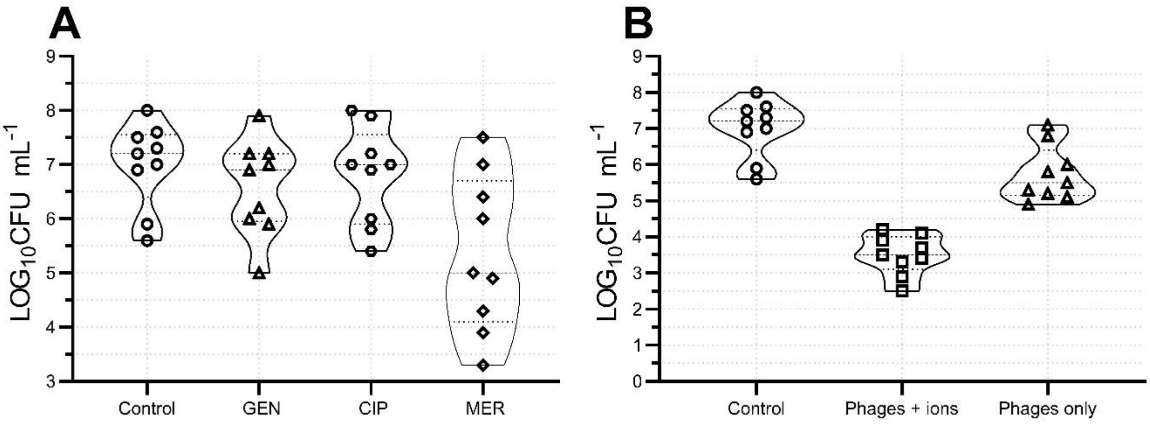

Figure S2. Antibiotic and Phage activity in the human heat inhibited plasma blood model. (A) Effectiveness of the *A. baumannii* treatment with antibiotics in the human heat inhibited plasma blood model. Control – without antibiotic addition; GEN - gentamicin, CIP – ciprofloxacin, MER – meropenem. (B) Influence of the ions reconstruction ($CaCl_2$, $FeSO_4$, or $MgCl_2$ ions were reconstructed at a final concentration of 5 mM) on the bacteriophage activity in the human heat inhibited plasma blood model

1. Wattam AR, Davis JJ, Assaf R, Boisvert S, Brettin T, Bun C, Conrad N, Dietrich EM, Disz T, Gabbard JL, Gerdes S, Henry CS, Kenyon RW, Machi D, Mao C, Nordberg EK, Olsen GJ, Murphy-Olson DE, Olson R, Overbeek R, Parrello B, Pusch GD, Shukla M, Vonstein V, Warren A, Xia F, Yoo H, Stevens RL. Improvements to PATRIC, the all-bacterial Bioinformatics Database and Analysis Resource Center. Nucleic Acids Res. 2017 Jan 4;45(D1):D535-D542. doi: 10.1093/nar/gkw1017. PMID: 27899627. PMCID: PMC5210524.
